# Process evaluation of the implementation of dementia-specific case conferences in nursing homes (FallDem): study protocol for a randomized controlled trial

**DOI:** 10.1186/1745-6215-15-485

**Published:** 2014-12-11

**Authors:** Daniela Holle, Martina Roes, Ines Buscher, Sven Reuther, René Müller, Margareta Halek

**Affiliations:** German Center for Neurodegenerative Diseases (DZNE), Stockumer Str. 12, 58453 Witten, Germany; School of Nursing Science, Witten/Herdecke University, Stockumer Str. 12, 58453 Witten, Germany

**Keywords:** Process evaluation, Stepped-wedged design, Implementation, Case conference, Dementia, Challenging behavior, Nursing home, mixed-method study

## Abstract

**Background:**

Challenging behaviors exhibited by individuals with dementia might result from an unmet need that they cannot communicate directly due to cognitive restrictions. A dementia-specific case conference represents a promising means of analyzing and exploring these unmet needs. The ongoing FallDem study is a stepped-wedged, cluster-randomized trial evaluating the effects of two different types of dementia-specific case conferences on the challenging behaviors of nursing home residents. This study protocol describes the process evaluation that is conducted, along with the FallDem study.

The goal of the process evaluation is to explain potential discrepancies between expected and observed outcomes, and to provide insights into implementation processes and recruitment strategies, as well as the contexts and contextual factors that promote or inhibit the implementation of dementia-specific case conferences.

**Methods/Design:**

The process evaluation will use a mixed-method design comprising longitudinal elements, in which quantitative and qualitative data will be gathered. Qualitative data will be analyzed using content analysis, documentary analysis and a documentary method. Quantitative data (standardized questionnaires) will be analyzed using descriptive statistics. Both types of data will complement one another and provide a more comprehensive picture of the different objects under investigation.

**Discussion:**

The process evaluation will allow for a comprehensive understanding of the changing processes and mechanisms underlying the ‘black box’ of the complex intervention of the FallDem study. These findings will provide practical knowledge regarding issues related to the implementation of dementia-specific case conferences in nursing homes.

**Trial registration:**

Current Controlled Trials identifier: ISRCTN20203855, registered on 10th July 2013.

## Background

Living with dementia is complicated by the presence of behavioral and psychological symptoms, which are sometimes also referred to as challenging behaviors [1]. People with dementia residing in nursing homes frequently exhibit these challenging behaviors [2], which include wandering, physical aggression, screaming, depression and resistance to receiving help with activities of daily living [3]. Rather than cognitive dysfunction, functional impairment or physical dependence, these behaviors constitute one of the greatest burdens for professional caregivers in nursing homes [4]. Challenging behaviors of people with dementia can have multiple etiologies that differ among individuals and settings. According to the need-driven dementia-compromised behavior (NDB) model, these behaviors result from an unmet need, and if they are responded to appropriately, patients’ quality of life will be enhanced [5]. A dementia-specific case conference represents a promising means of analyzing and exploring the unmet needs of people with dementia [6, 7]. Two different approaches to the dementia-specific case conference have been most commonly described in the literature [8]. The first approach entails the use of an assessment instrument to systematically guide nursing staff through the diagnostic process of a case conference (WELCOME-IdA; Innovative dementia-oriented Assessment) [9, 10]. The second approach involves the use of an open-thinking method to determine the potential triggers and causes of challenging behaviors rather than an assessment instrument (WELCOME-NEO) [11]. However, thus far, it is unclear whether the use of assessment instruments in dementia-specific case conferences are effective in supporting nursing teams in the diagnostic processes of need-driven behaviors [8]. Additionally, the advantages of dementia-specific case conferences using an open approach (without a standardized assessment) have not been explored in detail [12] or assessed in terms of their effectiveness.

The ongoing FallDem study (dementia-specific case conferences) is a stepped-wedged, cluster-randomized trial that aims to evaluate the uses of these two different types of dementia-specific case conferences (WELCOME-IdA and WELCOME-NEO) in 12 nursing homes [13]. This study is investigating whether these case conferences have an impact on residents´ challenging behaviors (primary outcome), quality of life and psychotropic drug use, as well as on the burnout and work-related stress experienced by nursing staff and their vocational action competences (secondary outcomes).

Because an effectiveness study is limited in its ability to provide information regarding whether an intervention is successful [14, 15], the FallDem study is being accompanied by a process evaluation, which is the main focus of the current study. A process evaluation provides insight into the so-called ‘black box’ of an effectiveness study and explains deviations between expected and observed outcomes. It further contributes to the understanding of how and to what extent an intervention is implemented in daily practice [16–19]. Furthermore, it provides information about the manner by which findings might be transferred across settings and populations [17, 20].

The process evaluation is of particular importance in the FallDem study because it is a dementia-specific case conference representing a complex intervention with interacting components [13] that will probably need time to be implemented in daily practice. It can be assumed that a dementia-specific case conference will have a delayed treatment effect [21] because teams in nursing homes have to learn how to employ it. This assumption needs to be further explored and defined in the process evaluation. Information about delayed treatment effects can subsequently aid in the design of a generalized linear mixed-effects model for the FallDem effectiveness study [13].

For the current study, the framework suggested by Grant *et al*. for designing process evaluations of cluster-randomized trials seems to be suitable because it considers ‘delivery to clusters’, the ‘responses of individuals’ and the ‘responses of clusters’ as essential domains to be studied [16]. These data can provide important evidence regarding the implementation success of an intervention [22]. Delivery to clusters includes information about how an intervention was delivered to each participating cluster and whether it was delivered as intended. Cluster variations in the delivery of an intervention might explain differences in its implementation and therefore variations in outcomes between clusters [16]. Response of individuals refers to the manners in which individuals in a target population react to a delivered intervention, for example, their attitudes, learning processes and behavioral changes [23]. If an intervention is not accepted by participants, the success of its implementation can be questioned [19]. Response of clusters describes how the intervention was adopted by each participating cluster. Differences in the adoption of clusters might also explain variations in outcomes [16].

The framework suggested by Grant *et al*. also includes the ‘recruitment of clusters’, ‘recruitment and reach of individuals’ and ‘context’ as areas to be studied in a process evaluation. These domains can help to assess the external validity of an effectiveness study [16]. Recruitment of clusters focuses on the strategies used by the research team to recruit clusters and the reasons why clusters decide (or not) to participate. Recruitment and reach of individuals describes the processes by which clusters identify and enroll individuals for an intervention and the proportion of recruited individuals who actually receive the intervention [16]. Information about recruitment and reach of clusters and individuals is important for deciding whether participants are representative of a target population [19]. A thorough description of the context in which a trial and intervention is embedded is of great importance for the generalization of findings, providing further relevant information about contextual factors that might act as barriers or facilitators to the implementation of the intervention [16, 18], thereby impeding or strengthening its effects [17]. In summary, the framework suggested by Grant *etal*. [16] combines domains that are important for evaluating the implementation success of an intervention or that are crucial for judging the external validity of an effectiveness study, explaining why this specific framework was used to design the process evaluation of the FallDem study.

**Figure 1:**
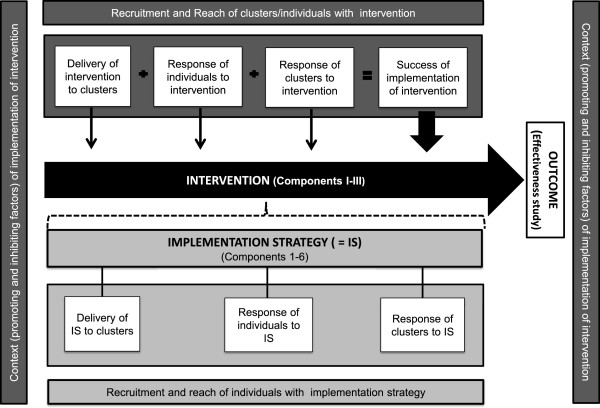
**Domains of process evaluation.**

Due to the complexity of dementia-specific case conferences, a comprehensive implementation strategy has been developed based on expert consultation to support the cluster-randomized controlled trial, in which case conferences are employed in daily practice. This strategy follows a step-wise plan and consists of the following six components: (1) information about the research project and dementia-specific case conferences, (2) kick-off meetings, (3) in-service training on dementia and moderator skills, (4) the establishment of a steering group, (5) reminders and (6) a telephone hotline (see Table 1). Although the effectiveness of the implementation strategy for the FallDem study will not be evaluated, knowledge about the usefulness of this strategy will be important for future implementations of dementia-specific case conferences in nursing homes. Therefore, four of the six domains of the process evaluation will be expanded to create a comprehensive implementation strategy, as shown in Figure 1.

**Table 1:** **Components of intervention and implementation strategies**

Components of intervention and implementation strategies		Content	Participants (target population)	Duration
Components of the intervention	I. In-service training in performing case conferences	▪ Aims and structures of CCs, NDB model, communication rules and use of IdA (only in the WELCOME-IdA intervention group)	▪ Manager of nursing home, head nurses of nursing teams and members of nursing teams (core team)	▪ Half a day
	II. Case conferences with support (training on the job)	▪ CCs are conducted with the support of trainers; the trainers assist the moderator, are involved in discussions and provide advice	▪ Members of nursing teams (core teams) and trainers	▪ 4 CCs within 3 months
	III. Case conferences without support	▪ CCs are conducted without aid	▪ Members of nursing teams (core teams)	▪ Minimum of 4 CCs within 4 months
Components of implementation strategy	(1) Information about project/case conferences	▪ Information concerning the project/intervention and data collection, time frame and organizational aspects	▪ Manager of nursing home, quality management and head nurses of nursing teams	▪ 3 hours
	(2) Kick-off meetings	▪ Information concerning project/intervention and data collection, time frame, and organizational aspects	▪ Nursing teams	▪ 1.5 hours
	(3) In-service training in a) dementia and challenging behaviors; and	▪ a) Diagnosis of dementia, forms and symptoms of dementia, causes of challenging behaviors and their management; and	▪ a) Nursing teams (core teams)	▪ a) Half a day
	b) Moderation of case conferences	▪ b) Training in moderation techniques	▪ b) 2 persons from each nursing team	▪ b) 2 days
	(4) Establishment of steering group	▪ Development of an implementation plan	▪ Manager of nursing home, quality management and members of both nursing teams (core teams)	▪ 2 days
	(5) Reminders	▪ Reminders via telephone to conduct CCs regularly	▪ All participants	▪ During intervention
	(6) Telephone hotline	▪ Questions concerning CCs	▪ All participants	▪ During intervention

Thus, thirteen research questions will guide the process evaluation, as shown in Table 2.

**Table 2:** **Research questions of process evaluation**

	Domain	Research question
Intervention	Delivery to clusters	1. Was the intervention delivered as intended to each nursing home (cluster)?
	Response of individuals	2. Which learning processes of the target population took place in response to the intervention?
		3. What is the attitude of the target population toward the intervention?
	Response of clusters	4. How was the intervention adopted by each nursing home (cluster)?
	Recruitment of cluster	5. How were nursing homes (clusters) sampled and recruited for the FallDem study?
		6. Why have the nursing homes participated (or not) in the FallDem study?
	Recruitment and reach of individuals	7. How were participants in the intervention recruited by the cluster, and which individuals in the target population actually received the intervention?
	Context	8. What is the context in which the intervention is being implemented?
		9. What contextual factors promoted or inhibited the implementation of the intervention?
Implementation strategy	Delivery to clusters	10. Was the implementation strategy delivered as intended for each nursing home (cluster)?
	Response of individuals	11. What is the attitude of the target population toward the implementation strategy?
	Response of cluster	12. How was the implementation strategy adopted by each nursing home (cluster)?
	Recruitment and reach of individuals	13. How were the participants in the implementation strategy recruited by the nursing homes (clusters), and which individuals in the target population actually received the implementation strategy?

## Methods/Design

### Study design

The process evaluation is a mixed-method study comprising longitudinal elements, for which quantitative and qualitative data are gathered (Tables 3 and 4). This method will be carried out alongside the FallDem study [13]) between September 2013 and March 2015. A total of 12 nursing homes (clusters) located in the area of North-Rhine-Westphalia (Germany) will take part in the process evaluation. The inclusion criteria for the clusters have been previously published elsewhere [13].

**Figure 2:**
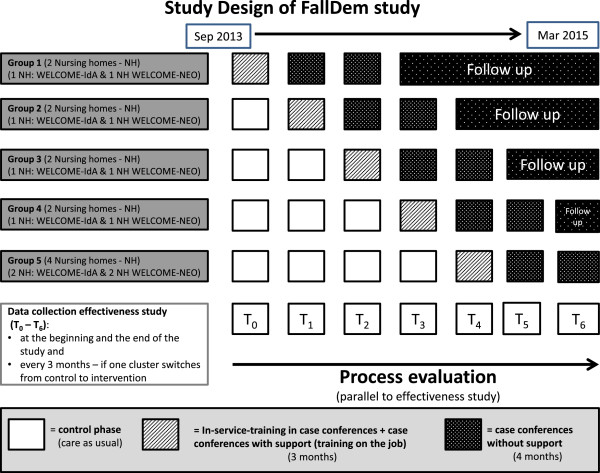
**Design of the FallDem study including process evaluation.**

In the FallDem study, the intervention is rolled out every three months to two nursing homes (cluster group) over a period of 18 months (stepped-wedge design). Within every cluster, two nursing wards, which each include one nursing team (core team), participate in the intervention. The order in which the 12 nursing homes will receive the intervention and the type of intervention are determined at random (six WELCOME-IdA clusters and six WELCOME-NEO clusters). Two nursing teams that belong to one cluster will receive the same type of intervention to avoid contamination between WELCOME-IdA and WELCOME-NEO. The last group of clusters includes four instead of two clusters because two nursing homes functioning as cluster reserves will be dropped before the end of the study (Figure 2).

The intervention phase (in-service training during case conferences, on-the-job training and case conferences with support) for each cluster lasts seven months, after which a follow-up phase, which lasts until the end of the data collection period for all 12 clusters (T6), occurs. Hence, the intervention phase is the same duration for each nursing home, but the durations of the pre-intervention and follow-up phases differ (Figure 2).

**Table 3:** **Data collection for domains of intervention**

	Research question	Data source		Informant of data collection	Procedure of data collection	Time of data collection
Delivery of clusters (intervention)	1. Was the intervention delivered as intended to each nursing home (cluster)?	A	attendance lists of in-service training in performing case conferences (I), training on the job (II) and case conferences without support (III)	-	proxy: assessed by trainers of project team	during in-service training, trainings on the job and case conferences without support
		B	standardized protocols of dementia-specific case conferences	-	documented by keeper of the minutes of case conference	during on-the-job training and case conferences without support
		C	written documentation of in-service training in performing case conferences (I) and training on the job (II) (deviation from curriculum)	trainers	documented by trainers of project team	after in-service training and on-the-job training
Response of individuals (intervention)	2. Which learning processes of the target population took place in response to the intervention?	D	audiotape of case conferences for four nursing teams (n = 24/6 per team); 2 teams using WELCOME-NEO, and 2 teams performing WELCOME-IdA	-	audiotaped by project team	during 2 on-the-job trainings and 4 case conferences without support
	3. What is the attitude of the target population toward the intervention?	E	standardized questionnaire to assess attitudes toward case conferences	nursing teams	self-assessed by nursing teams	T0 to T6
		F	standardized questionnaire to evaluate in-service training in performing case conferences (I) and training on the job (II)	nursing teams	self-assessed by participants	after in-service training and on-the-job training
		G	semi-structured telephone interviews to evaluate case conferences (n = 96/4 per nursing team)	head nurses	interviewed by project team	during intervention phase
		H	semi-structured group interview to evaluate intervention (n = 12)	moderators	interviewed by project team	at end of intervention phase
		I	semi-structured group interviews to evaluate intervention (n = 24, 2 per cluster)	2 core teams	interviewed by project team	at end of intervention phase
Response of cluster (intervention)	4. How was the intervention adopted by each nursing home (cluster)?	G	semi-structured telephone interviews to evaluate case conferences (n = 96/4 per nursing team)	head nurses	interviewed by project team	during intervention phase
		I	semi-structured group interviews to evaluate case conferences (n = 24/1 per team)	2 core teams	interviewed by project team	at end of intervention phase
		J	semi-structured group interview to evaluate case conferences (n = 12/1 per cluster)	steering groups	interviewed by project team	at end of intervention phase
		H	semi-structured group interview to evaluate case conferences (n = 12/1 per cluster)	moderators	interviewed by project team	at end of intervention phase
Recruitment of cluster (intervention)	5. How were the nursing homes (clusters) sampled and recruited for the FallDem study?	N	written documentation of recruitment procedure	project team	project team	during recruitment of cluster
	6. Why have the nursing homes participated (or not) in the FallDem study?	K	semi-structured telephone interviews to assess ‘care as usual’ (n = 24/1 per team)	head nurses	interviewed by project team	at baseline (T0)
		I	semi-structured group interviews (n = 24/1 per team)	2 core teams	interviewed by project team	at end of intervention phase
		J	semi-structured group interview (n = 12/1 per cluster)	steering groups	interviewed by project team	at end of intervention phase
Recruitment and reach of individuals (intervention)	7. How were the participants of the intervention recruited by the cluster, and who in the target population actually received the intervention?	A	attendance lists of in-service training in performing case conferences (I), training on the job (II) and case conferences without support (III)	-	proxy: assessed by trainers of project team	during in-service trainings, on-the-job trainings and case conferences with support
		J	semi-structured group interview (n = 12)	steering groups	interviewed by project team	at end of intervention phase
		H	semi-structured group interview (n = 12)	moderators	interviewed by project team	at end of intervention phase
Context (intervention)	8. What is the context in which the intervention is being implemented?	K	semi-structured telephone interviews to assess ‘care as usual’ (n = 24)	head nurses	interviewed by project team	at baseline (T0)
		L	Dementia Milieu Assessment (DMA)	-	proxy: assessed by project team	T0 and T6
		M	standardized questionnaire to assess organizational and structural characteristics of nursing home/nursing wards	manager/head nurses	self-assessed by manager of nursing homes/head nurses of nursing wards	T0 to T6
	9. What contextual factors promote or inhibit the implementation of the intervention?	A-N	all data assessed throughout process evaluation	-	-	-

**Table 4:** **Data collection for domains of implementation strategy**

	Research question	Data source		Informant of data collection	Procedure of data collection	Time of data collection
Delivery of clusters (implementation strategy)	10. Was the implementation strategy delivered as intended to each nursing home (cluster)?	A	attendance lists of in-service trainings (3a, b) and steering group meetings (4)	-	proxy: assessed by teachers of project team	during training and meetings
		C	written documentation of in-service training (3a, b) and steering group meetings (4) (deviation from curriculum)	trainers	documented by project team	after training and meetings
Response of individuals (implementation strategy)	11. What is the attitude of the target population toward the implementation strategy?	F	standardized questionnaire to evaluate in-service training (3a, b) and steering group meetings (4)	nursing teams	self-assessed by participants	after training and meetings
		G	semi-structured telephone interviews to evaluate implementation strategy (n = 96/4 per nursing ward)	head nurses	interviewed by project team	during intervention phase
		I	semi-structured group interviews to evaluate case conferences (n = 24/1 per team)	core teams	interviewed by project team	at end of intervention phase
		J	semi-structured group interviews to evaluate case conferences (n = 12/1 per cluster	steering groups	interviewed by project team	at end of intervention phase
		H	semi-structured group interview to evaluate case conferences (n = 12/1 per cluster)	moderators	interviewed by project team	at end of intervention phase
Response of cluster (implementation strategy)	12. How was the implementation strategy adopted by each cluster?	G	semi-structured telephone interviews to evaluate implementation strategy (n = 96/4 per nursing ward)	head nurses	interviewed by project team	during intervention phase
		I	semi-structured group interviews to evaluate case conferences (n = 24/1 per team)	2 core teams	interviewed by project team	at end of intervention phase
		J	semi-structured group interview to evaluate case conferences (n = 12/1 per cluster)	steering groups	interviewed by project team	at end of intervention phase
		H	semi-structured group interview to evaluate case conferences (n = 12/1 per cluster)	moderators	interviewed by project team	at end of intervention phase
Recruitment and reach of individuals (implementation strategy)	13. How were participants of the implementation strategy (IS) recruited by the nursing homes (clusters), and who in the target population actually received the IS?	A	attendance lists of in-service training (3a, b) and steering group meetings (4)	-	proxy: assessed by teachers of project team	during training, meetings
		H	semi-structured group interview (n = 12)	steering groups	interviewed by project team	at end of intervention phase

### Intervention

**Figure 3:**
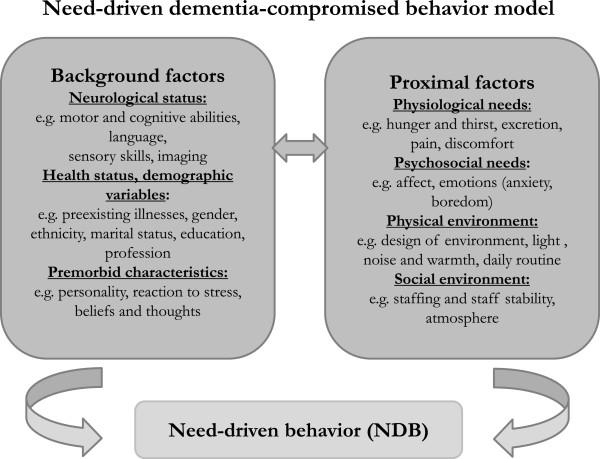
**Need-driven dementia-compromised behavior model.**

Dementia-specific case conferences are defined as structured, goal-directed, intra-professional conversational procedures that support the nursing staff in the descriptions and analyses of triggers and the causes of residents’ challenging behaviors [6]. Dementia-specific case conferences are embedded within the general theory of hermeneutics and the NDB model [24, 25]. Hermeneutics generally describes the philosophy of understanding and interpreting the social actions of individuals, groups and organizations [26]. Furthermore, using hermeneutics as a strategy to interpret observed behaviors strengthens the abilities of nursing teams to understand the perspectives of people with dementia with respect to their social or biographical backgrounds [24, 27]. The NDB model applies a more specific theory that provides explanations for the reasons underlying the challenging behaviors exhibited by individuals with dementia. In the NDB model, challenging behaviors reflect the interactions of relatively stable background factors (such as neurological factors, health status, demographic and pre-morbid characteristics) with more changeable proximal factors (such as physiological and psychological needs and physical and social environments) that commonly result in need-driven behaviors [5] (Figure 3). Thus, the aim of a dementia-specific case conference is to identify and analyze those background and proximal factors that commonly cause challenging behaviors in people with dementia.

WELCOME-IdA is a dementia-specific case conference that includes a comprehensive assessment system called IdA (Innovative dementia-oriented Assessment) [28]. IdA is based on the NDB model and comprises a two-step procedure. In the first step, the nursing staff is guided through a thorough description and quantification of a challenging behavior. In the second step, the nursing staff is guided through a search for potential triggers and causes of the challenging behavior. For this second step, IdA provides five dimensions (‘state of health and independence in everyday life’, ‘communication’, ‘personality and lifestyle before the onset of dementia’, ‘mood and emotions’ and ‘environmental influences’) with specific guiding questions (Table 5).

**Table 5:** **Process structure and key characteristics of both dementia-specific case conferences**

	WELCOME-IdA	WELCOME-NEO
Process structure	Preparation of case conferences (CCs)	
	Introduction (welcome, time frame and roles)	
	▪ Description/quantification of challenging behavior on the basis of IdA (14 guiding questions)	▪ Description/quantification of challenging behavior without assessment (narrative)
	▪ Analyses of triggers and causes of challenging behavior on the basis of IdA (48 guiding questions)	▪ Analyses of triggers and causes of challenging behavior without assessment (narrative)
	Planning of care intervention based on analysis of situation	
	Closing (for example, personal reflection, what have I learned from the case?)	
	Post-processing of case conference (for example, responsibility to transfer results to daily care routines)	
	Evaluation of case conference (for example, changes in challenging behavior due to care interventions and adoption of care interventions)	
	WELCOME-IdA and WELCOME-NEO	
Key characteristics	Participants (core team)	
	1 moderator	
	1 keeper of minutes	
	1 case reporter	
	2 to 5 reflection partners	
	Location	
	undisturbed room/area	
	Duration	
	60 to 90 minutes	
	Intervals	
	(at least) monthly	

WELCOME-NEO is a dementia-specific case conference that supports an open-thinking method. Therefore, it requires that the description and analysis of a challenging behavior be provided in a narrative manner, instead of relying on an assessment instrument for its description and quantification, as well as the determination of its potential triggers and causes (Table 5). Both types of dementia-specific case conferences have predefined process structures and key characteristics that are shown in Table 5.

The interventions of these two dementia-specific case conferences will begin with training in the respective model (WELCOME-IdA or WELCOME-NEO) and will be followed by four supported case conferences (on-the-job training). Then, a minimum of four dementia-specific case conferences will be performed without any assistance (case conferences without support) by the nursing teams (Table 1).

### Implementation strategy

The comprehensive implementation strategy will follow a gradual plan consisting of six components as follows:

1. Information about the research project and dementia-specific case conferences: the implementation strategy will start with a detailed meeting (three hours) with representatives of the top management, quality management and nursing teams of each nursing home to discuss topics, including the key elements of the research project and dementia-specific case conferences.
2. Kick-off meetings: following the meeting with the leaders, a kick-off meeting (one and a half hours) will be organized at each nursing home (cluster) to provide information to the participating nursing teams about the research project and dementia-specific case conferences. A direct strategic communication to staff members of the nursing homes will demonstrate that the researchers value the decision to participate in the research project [29]. These meetings will be held with the general understanding that the implementation of the intervention requires communication through existing organizational channels and, over time, among the members of the nursing home staff [30].
3. In-service training: the in-service training will comprise two modules, including ‘dementia and challenging behavior’ and ‘moderator skills’. Previous studies have shown that significant knowledge about dementia and challenging behaviors as well as moderator skills are essential for the successful implementation of dementia-specific case conferences [7, 10].
  - Dementia and challenging behaviors (half a day):
    participants: nursing teams (core teams) (open to all staff and guests of the nursing home)
    aims: i) participants develop an emphatic attitude towards individuals with dementia and use their new knowledge to gain a better understanding about dementia and associated behaviors; and ii) participants distinguish between different forms and stages of dementia and differentiate between dementia and other syndromes, such as delirium and depression.

(b) Moderation skills (two days)
  participants: two persons per nursing team (four moderators per cluster)
  aims: participants learn the importance and tasks of a moderator in a case conference; basic communication skills that promote a respectful work environment are reviewed; and participants learn moderation skills and how to handle difficult situations during moderation.
  Knowledge plays a major role in the implementation of concepts; for example, in the FallDem study, knowledge is circulated [31] among the participating nursing homes, necessitating knowledge brokers [32]. Knowledge circulation refers to the process of transferring research knowledge to practice and is related to the institutionalization of an intervention [33]. Moderators also assists nursing teams by drawing analogic links between solutions determined by reflecting on past cases [34].
(4) Establishment of a steering group:
  Each nursing home (cluster) will form a steering group. The steering group should consist of representatives from top management, quality management and members of both nursing teams (core teams). This group will be responsible for the implementation process (such as the designation of responsibilities and creation of structural requirements). It will also be responsible for conducting an assessment of the strengths and weaknesses of the organization in relation to the implementation context. This assessment is important for gaining information about the ‘implementation climate’ [29]. Based on these results, a specific implementation plan will be developed. Importantly, the steering group will also analyze the impact of the FallDem study on organizational processes [29]. Usually, the following two questions are of interest: ‘How relevant is the intervention dementia-specific case conference to the nursing homes?’ and ‘How much will this improve performance quality and outcome?’ [35]. The steering group will meet at least three times and will be moderated by a professional external trainer.
(5) Reminders:
  Reminders will be used as an additional means of supporting the implementation process. They will be conducted via telephone prior to the dementia-specific case conferences. In total, four reminders will be given per nursing team (one per month) during the seven months of the intervention phase. They will support the adoption process on the individual level and on the organizational level [35].
(6) Telephone hotline:
  A telephone hotline will be established to ensure prompt help with practical difficulties encountered during the organization and performance of the dementia-specific case conferences. This hotline will be maintained throughout the intervention phase. During the phase in which the core team members and moderator act, problems might occur, particularly when they conduct case conferences on their own. The challenge lies in the assumption that the case conference can be conducted according to the study protocol, but in-action heuristic patterns and default options may influence the adoption process [35].

### Data collection

For the process evaluation, a combination of qualitative and quantitative data is gathered that can be subdivided into 14 different data sources (A to N). Table 3 summarizes all of the data that will be assessed in the process evaluation with regard to the intervention. It further provides information about the informant, procedure and time of data collection.

#### Delivery of intervention to clusters

The delivery of the intervention to the 12 clusters will be assessed using standardized attendance lists (data source A). The individuals (name, profession and function) participating in the different components of the intervention (I to III) will be documented. Moreover, nonconformities to the training curricula (I to II) will be documented. Deviations will be recorded in relation to the participants, durations, didactic methods and contents of the training units (data source C). Finally, standardized protocols will be written to document each case conference, providing further information regarding their process structures and key characteristics (data source B).

#### Response of individuals to intervention

The response of individuals toward the dementia-specific case conferences will be analyzed in relation to the learning processes of the core teams. A total of 24 dementia-specific case conferences in a subsample of four nursing teams will be audiotaped (six per team) (data source D) [36]. Two of the selected nursing teams will conduct WELCOME-IdA (12 audiotapes), and the other two will conduct WELCOME-NEO (12 audiotapes). The selection criterion for these four teams will be prior experience in conducting case conferences. The two nursing teams with the most and the two with the least prior experience in conducting case conferences will be selected (also see data source K).

The attitudes of individuals toward the intervention will be assessed first, using a standardized questionnaire (data source E) containing 11 statements pertaining to the use of dementia-specific case conferences. It will be administered to the nursing teams to document changes in attitude toward the intervention throughout the FallDem study (T0 to T6). Each statement will comprise four responses (from 1 = totally disagree to 4 = totally agree). Second, a standardized evaluation sheet will be provided to the participants of the training (I and II) at the end of each session (data source F).

Moreover, four semi-structured telephone interviews [37] with the head nurse of each nursing team (data source G) will be carried out to gain insight into the overall attitudes of the target populations toward the intervention. Finally, semi-structured group interviews [38] will be conducted with all moderators and all 24 nursing teams at the end of the intervention phase (data sources H and I).

#### Response of cluster to intervention

For the response of the clusters toward the intervention, four semi-structured telephone interviews [37] will be conducted with the head nurses of each participating nursing ward during the intervention phase. The head nurse should take part in the dementia-specific case conferences (data source G). The goal of the interviews with the head nurse will be to assess whether the structured preparation and post-processing of the dementia-specific case conferences have taken place and whether any changes have occurred due to their implementation. Finally, semi-structured group interviews [38] will be conducted with the steering group, moderators and nursing teams (core teams) at the end of the intervention phase to gain perspective about the overall response of the cluster toward the intervention (data sources H, I and J).

#### Recruitment of cluster

To gain insight into the recruitment of the cluster, the recruitment processes of the nursing homes will be documented by the project team (data source N). Additionally, semi-structured telephone interviews [37] will be conducted with each head nurse of the 24 participating nursing wards at the beginning of the FallDem study (data source K). The telephone interviews will provide information regarding the reason why each ward has decided to take part in the FallDem study. In addition, semi-structured group interviews [38] with each steering group (data source J) and each nursing team (core teams) (data source I) will be conducted at the end of the intervention phase to explore in depth the motivations of each cluster and each ward for participating in the FallDem study.

#### Recruitment and reach of individuals with intervention

The recruitment and reach of individuals phase of the intervention will be explored by conducting semi-structured group interviews [38] with the steering groups and moderators at the end of the intervention phase (data sources H and J). The interviews will address the criteria that clusters have used to allocate core team members. Moreover, attendance lists will be used to assess the individuals in the target population who actually received the different components of the intervention (I to III) (data source A).

#### Context of intervention

The context in which the intervention is implemented will first be assessed through a semi-structured telephone interview [37] with the head nurse of each participating nursing ward at the beginning of the FallDem study (data source K). The telephone interview will aim to explore whether nursing teams already have experience in performing dementia-specific case conferences and, if they do, which factors promote or inhibit their performance. Second, the public area of each participating nursing ward will be evaluated with the Dementia Milieu Assessment (DMA) [39]. The DMA assesses the ‘dementia-friendliness of environments’ and will be conducted during a two-hour observation period from 3 to 5 pm. The DMA will be used at the beginning and end of the FallDem study (data source L). Third, a standardized questionnaire will be used quarterly (T0 to T6) to assess the organizational characteristics of the 12 participating clusters and 24 participating nursing wards (such as the size, number of employees, types of living arrangements, staff specialization and characteristics of residents) (data source M).

To gain insight into the contextual factors that promote or inhibit the implementation of dementia-specific case conferences, all data sources of the process evaluation (A to N) will be used and secondarily analyzed. No additional data will be gathered to answer this research question. For the process evaluation of the implementation strategy, seven different data sources (A, C, F, G, H, I and J), which are summarized in Table 4, will be used.

#### Delivery of implementation strategy to clusters

The delivery of the implementation strategy to the 12 clusters will be assessed using standardized attendance lists (data source A). The individuals (name, profession and function) participating in the steering group meetings and additional in-service training will be documented (3a, b). Moreover, nonconformities to the meeting curricula of the steering group and additional in-service training will be documented. Deviations will be recorded in relation to the participants, durations, didactic methods and contents of the training units (data source C).

#### Response of individuals to implementation strategy

The attitudes of individuals toward the implementation strategy will be assessed first using standardized evaluation sheets that will be distributed to the participants at the steering group meeting (2) and the additional in-service training (3a, b) at the end of each session (data source F). Moreover, four semi-structured telephone interviews [37] with the head nurse of each nursing team (data source G) will be carried out to gain insight into the overall attitudes of the target populations toward the implementation strategy. Finally, semi-structured group interviews [38] will be conducted with all of the moderators, the steering group and all 24 nursing teams at the end of the intervention phase (data sources H, I and J).

#### Response of cluster to implementation strategy

For the response of the clusters toward the implementation strategy, four semi-structured telephone interviews [37] will be conducted with the head nurses of each participating nursing ward during the intervention phase. The head nurse should take part in the steering group meeting (data source G). Therefore, the interviews with the head nurse will aim to assess whether the steering group has supported the implementation process of the dementia-specific case conferences and assisted the nursing teams in implementing them in their daily routines. Finally, semi-structured group interviews [38] will be conducted with the steering group, the moderators and the nursing teams (core teams) at the end of the intervention phase to evaluate the overall response of the cluster to the implementation strategy (data sources H, I and J).

#### Recruitment and reach of individuals with implementation strategy

The recruitment and reach of individuals with the implementation strategy will be explored using semi-structured group interviews [38] with the steering groups (data sources J). The interviews will address the criteria that clusters have used to allocate the members of the steering group and the moderators. Moreover, standardized questionnaires will be used to evaluate who in the target population actually took part in the steering group meetings and additional in-service trainings (3a, b) (data source F).

All semi-structured telephone and group interviews, as well as the audiotapes of the dementia-specific case conferences, will be transcribed verbatim by a professional translation agency and subsequently proofread by one member of the research team prior to data analysis. For the transcription of the interviews, the method of Kuckartz [40] will be used.

### Data analysis

The FallDem study will be evaluated using a mixed-method design comprising qualitative and quantitative data, which will complement each other, providing a more comprehensive picture of the different concepts under investigation [41]. Guest [42] has noted that consideration of the point of interface (the integration of qualitative and quantitative data) leads to the perspective that ‘the timing and the purpose of data integration’ are the most important:

‘The timing of integration is critically important because it not only conveys when data sets are used with respect to one another but also whether the data sets depend on each other. The purpose of integration denotes the reason for connecting or mixing data sets at each stage of the research process’ ([42] page 147).

Bazeley and Kemp have argued that there must be ‘interdependence of component approaches during the analytic writing process (…)’ ([43] page 69). Simply preparing final conclusions on the basis of different datasets does not mean that the methods have been integrated. Therefore, integration must occur before conclusions are drawn, the results of which will represent ‘something that would not have been available without that integration’ ([43] page 69).

In this study, data integration will take place on two levels. First, data within the same domain of the process evaluation will be combined to obtain a comprehensive picture of each domain and across domains. For this purpose, integrated analysis will be performed using a DNA double helix method, which compromises sense and antisense strands [44]. Expecting divergent results (within and across domains), this strategy will allow for the determination of concordant findings (the sense strand of the analysis; based on previous studies and expected results) and the consideration of antisense or dissonant findings as counterpoints (the findings of the current FallDem study):

‘The divergence of findings can then be used as a promoter (and) can continue in a series of iterations. (…) Thus, reconciliation is sought by undertaking analysis that facilitates a continuous dialogue or exchange of multiple data to understand the phenomena of interest’ ([43] page 67f).

Second, data from the three domains, including the delivery of the intervention to the cluster, response of individuals to the intervention and response of cluster to the intervention, will be integrated in the statistical analysis of the FallDem effectiveness study through blendin*g*[43], meaning that a new variable (time combined with the success of the implementation) will be integrated in the generalized linear mixed-effects model [13]. To achieve this aim, the integration of the data will also be performed in a transformative manner using quantized qualitative coding (the success of the implementation), which can be analyzed in relation to each cluster (also see defining the delayed treatment effect) (Tables 6 and 7).

**Table 6:** **Data analysis of domains of intervention**

Domain	Research question	Data source		Theoretical basis	Method of data analysis	Method of data integration
Delivery of clusters (intervention)	1. Was the intervention delivered as intended for each nursing home (cluster)?	A	attendance lists of in-service training in performing case conferences (I), training on the job (II) and case conferences without support (III)	▪ Case conference model (key characteristics)	▪ Descriptive statistics [45]	▪ DNA double helix
						▪ Blending
						▪ Transformation
		B	standardized protocols of dementia-specific case conferences	▪ Case conference model (process structure and key characteristics)	▪ Documentary analysis [46]	
		C	written documentation of in-service training in performing case conferences (I)and training on the job (II) (deviation from curriculum)	▪ Curriculum (in-service training in performing case conferences (I) and on-the-job training (II))	▪ Documentary analysis [46]	
Response and reach of individuals (intervention)	2. Which learning processes of the target population took place in response to the intervention?	D	audiotapes of case conferences of four nursing teams (n =24/6 per team); 2 teams using WELCOME-NEO and 2 teams performing WELCOME-IdA	▪ Act for teams, Kasseler competence inventory [47]	▪ Documentary method [48]	-
	3. What is the attitude of the target population toward the intervention?	E	standardized questionnaire to assess attitudes towards case conferences	▪ Adoption model [49]	▪ Descriptive statistics [45]	▪ DNA double helix
						▪ Blending
		F	standardized questionnaire to evaluate in-service training in performing case conferences (I) and training on the job (II)	▪ Curriculum (in-service training in performing case conferences (I) and on-the-job training (II)	▪ Descriptive statistics [45]	▪ Transformation
		G	semi-structured telephone interviews to evaluate case conferences (n = 96/6 per nursing team)	▪ Adoption model [49]	▪ Content analysis [50]	
		H	semi-structured group interviews to evaluate intervention (n = 12)	▪ Adoption model [49]	▪ Content analysis [50]	
		I	semi-structured group interviews to evaluate intervention (n = 24, 2 per cluster)	▪ Adoption model [49]	▪ Content analysis [50]	
Response of cluster (intervention)	4. How was the intervention adopted by each nursing home (cluster)?	G	semi-structured telephone interviews to evaluate case conferences (n = 96/6 per nursing team)	▪ Adoption model [49]	▪ Content analysis [50]	▪ DNA double helix
						▪ Blending
						▪ Transformation
		I	semi-structured group interviews to evaluate case conferences (n = 24/1 per team)	▪ Adoption model [49]	▪ Content analysis [50]	
		J	semi-structured group interview to evaluate case conferences (n = 12/1 per cluster)	▪ Adoption model [49]	▪ Content analysis [50]	
		H	semi-structured group interview to evaluate case conferences (n = 12/1 per cluster)	▪ Adoption model [49]	▪ Content analysis [50]	
Recruitment of cluster (intervention)	5. How were the nursing homes (clusters) sampled and recruited for the FallDem study?	N	written documentation of recruitment procedure	-	-	-
	6. Why have the nursing homes participated (or not) in the FallDem study?	K	semi-structured telephone interviews to assess ‘care as usual’ (n = 24/1 per team)	-	▪ Content analysis [50]	▪ DNA double helix
		I	semi-structured group interviews (n = 24/1 per team)	-	▪ Content analysis [50]	
		J	semi-structured group interviews (n = 12/1 per cluster)	-	▪ Content analysis [50]	
Recruitment and reach of individuals (intervention)	7. How were the participants of the intervention recruited by the cluster, and who in the target population actually received the intervention?	A	attendance lists of in-service training in performing case conferences (I), training on the job (II) and case conferences without support (III)	▪ Case conference model (key characteristics)	▪ Descriptive statistics [45]	▪ DNA double helix
		J	semi-structured group interview (n = 12)	-	▪ Content analysis [50]	
		H	semi-structured group interview (n = 12)	-	▪ Content analysis [50]	
Context (intervention)	8. What is the context in which the intervention is being implemented?	K	semi-structured telephone interviews to assess ‘care as usual’ (n = 24)	▪ Case conference model (process structure and key characteristics)	▪ Content analysis [50]	▪ DNA double helix
		L	Dementia milieu assessment (DMA)	-		
		M	standardized questionnaire to assess organizational and structural characteristics of nursing homes/nursing wards	▪ Consolidated Framework for Implementation Research (CFIR) [51, 52]	▪ Descriptive statistics [45]	
	9. What contextual factors promote or inhibit the implementation of the intervention?	A-M	All data assessed throughout the evaluation process	▪ CFIR [51, 52]	▪ Content analysis [50]	▪ DNA double helix

**Table 7:** **Data analysis of domains of implementation strategy**

Domain	Research question	Data source		Theoretical basis	Method of data analysis	Method of data integration
Delivery of clusters (implementation strategy)	10. Was the implementation strategy delivered as intended for each nursing home (cluster)?	A	attendance lists of in-service training (3a, b) and steering group meetings (4)	-	▪ Descriptive statistics [45]	▪ DNA double helix
		C	written documentation of in-service training (3a, b) and steering group meetings (4) (deviation from curriculum)	▪ Curriculum (in-service training in dementia and moderator skills (3a, b) and establishment of steering group )	▪ Documentary analysis [46]	
Response of individuals (implementation strategy)	11. What is the attitude of the target population toward the implementation strategy?	F	standardized questionnaire to evaluate in-service trainings (3a, b) and steering group meetings (4)	-	▪ Descriptive statistics [45]	▪ DNA double helix
		G	semi-structured telephone interviews to evaluate implementation strategy (n = 96/4 per nursing ward)	▪ Adoption model [49]	▪ Content analysis [50]	
		I	semi-structured group interviews to evaluate case conferences (n = 24/1 per team)	▪ Adoption model [49]	▪ Content analysis [50]	
		J	semi-structured group interviews to evaluate case conferences (n = 12/1 per cluster)	▪ Adoption model [49]	▪ Content analysis [50]	
		H	semi-structured group interview to evaluate case conferences (n = 12/1 per cluster)	▪ Adoption model [49]	▪ Content analysis [50]	
Response of cluster (implementation strategy)	12. How was the implementation strategy adopted by each cluster?	G	semi-structured telephone interviews to evaluate implementation strategy (n = 96/4 per nursing ward)	▪ Adoption model [49]	▪ Content analysis [50]	▪ DNA double helix
		I	semi-structured group interviews to evaluate case conferences (n = 24/1 per team)	▪ Adoption model [49]	▪ Content analysis [50]	
		J	semi-structured group interview to evaluate case conferences (n = 12/1 per cluster)	▪ Adoption model [49]	▪ Content analysis [50]	
		H	semi-structured group interview to evaluate case conferences (n = 12/1 per cluster)	▪ Adoption model [49]	▪ Content analysis [50]	
Recruitment of individuals (implementation strategy)	13. How were participants of the implementation strategy (IS) recruited by the nursing homes, and who in the target population actually received the IS?	A	attendance lists of in-service training (3a, b) and steering group meetings (4)	-	▪ Descriptive statistics [45]	▪ DNA double helix
		H	semi-structured group interview (n = 12)	-	▪ Content analysis [50]	

All quantitative data (standardized questionnaire) will be analyzed using descriptive statistics [45]. All qualitative data will be analyzed using content analysis [50, 53], a documentary method [48, 54, 55] or document analysis [46].

The semi-structured telephone and group interviews will be primarily deductively assessed using the content analysis technique of Mayring [50, 53]. The application of the deductive category employs previously formulated, theoretically derived aspects of an analysis (see Tables 6 and 7; theoretical basis) and connects these aspects with the text. In a methodological, controlled assignment, a passage of text is linked with a category. Each deductive category has an explicit definition, example and coding rule with regard to the theory and material and is revised during the analysis [50, 53].

The audiotapes of the dementia-specific case conferences will be analyzed using the documentary method [48, 54, 55], which is a reconstructive analysis tool that allows for case comparisons (key concept). Thus, internal and cross-case comparisons as well as the determination of the comparative knowledge of an interviewer will be possible. Four phases will be implemented as follows: (1) the formation of an outline and detailed paraphrasing that begins prior to transcription with a focus on ‘what is said’. To avoid bias, the whole case conference will be transcribed; (2) reflection on interpretation, examining the reconstruction and explication of the orientation frame with a focus on ‘how it is said’ (during phase 2, internal and cross-case comparisons will be initiated to ensure inter-subjectivity of the results); and (3) the ‘stage of case description’ (case structure) phase, during which the essential reconstructed elements will be summarized. For phase 1 (what is said), the ‘act for team model’ will be used [47]. The standardized protocols for the dementia-specific case conferences and the written documentation will be analyzed using documentary analysis [46] (see Tables 6 and 7).

#### Defining delayed treatment effect

It can be assumed that a dementia-specific case conference shows its full treatment effect (100%) when it is delivered over time as intended, is accepted by the nursing team and is adopted by the nursing home. The earliest time point at which a full effect could be expected according to the data is at the transition from training on the job (II) to case conferences without support (III). However, case conferences represent complex interventions that require training and education (as expected, the staff may need more time to learn how to use the case conferences in their daily work routines). Therefore, a delayed treatment effect cannot be excluded (for example, the intervention being only 75% effective after transition from training on the job (II) to case conferences without support (III)). This information provided by the process evaluation will be important for improving model building using a generalized linear mixed-effects model of the effectiveness study. The delay must be modeled as a fractional treatment effect in the mixed-effects model as described by Hussey and Hughes [21]. To define a possible delayed treatment effect for each nursing home, the data pertaining to the delivery of intervention to clusters, the response of individuals to the intervention and the response of clusters to the intervention will be used.

### Ethical considerations

The Institutional Review Board for Ethics in Research, German Society for Science in Nursing (E-DG-P) has discussed and considered the proposal, entitled Fallbesprechungen bei Menschen mit Demenz (FallDem), Teil II: Interventionsdurchführung (delivered in August 2011), and has approved this study. Informed consent will be obtained from each participant before the start of the trial.

## Discussion

The process evaluation of the FallDem study will provide insights into the implementation process of dementia-specific case conferences via a cluster-randomized study and help to define the overall success of the implementation. Implementation errors can be explained in cases in which the results of the effectiveness study show no effect or weak effects [56]. Thus, this study overcomes the general criticism that implementation issues have been overlooked in healthcare research on psychosocial interventions in daily residential dementia care [57].

Testing a clinical intervention while gathering information on its implementation in a real-world situation, which is also called an effective-implementation type I hybrid design [58], can further facilitate the incorporation of a new innovation into daily practice [39]. For this study, knowledge about contextual factors that might promote or inhibit the implementation of the dementia-specific case conferences and information about the delivery, acceptability and adoption of the implementation strategy will be gathered. Both types of data will be subsequently used to strengthen the implementation strategy, which in turn will facilitate and reinforce the implementation of the dementia-specific case conferences in daily routines in nursing homes once the stepped-wedged cluster-randomized study has been completed. The results will be further used to design a successive hybrid III study [58], in which the implementation strategy will be tested and information on the clinical intervention and its related outcomes will be gathered.

Using the results of the process evaluation to design the generalized mixed-effects model of the stepped-wedged, cluster-randomized effectiveness study, new methodological pathways in healthcare research will be explored that have not been widely used to date. Previous studies, such as those on the effects of depression and behavior management programs on nursing home residents with dementia [59, 60], have considered a full treatment effect directly after the intervention has been rolled out to the cluster. Due to the complexities of both programs, a delayed treatment effect can also be assumed in both of these studies, which may have affected their results.

The limitations of the process evaluation of the FallDem study are also worth noting. The maintenance of dementia-specific case conferences in nursing is an important issue to be explored in a process evaluation; this issue will be only partially addressed in this study. The manner by which individuals and organizations respond to and adopt an intervention may change over time [16], thus, although that topic is beyond the scope of the present study, it may be interesting to determine what will happen during the follow-up-period after the intervention phase of the FallDem study.

## Trial status

The trial was initiated in 2013 and will be completed by the end of 2015. The recruitment of the nursing homes was completed in the fall of 2013. The recruitment of the participating staff will be completed by the end of 2014. Results will be reported at the ends of 2015 and 2016.

## Abbreviations

CC: Case conferences
CFIR: Consolidated Framework for Implementation Research
DMA: Dementia Milieu Assessment
FallDem: Fallbesprechungen bei Demenz (dementia-specific case conferences)
IdA: Innovative dementia-oriented Assessment
IS: Implementation strategy
NDB: Need-driven dementia-compromised behavior
WELCOME-IdA: Witten model of case conferences for people with dementia, the Innovative dementia-oriented Assessment
WELCOME-NEO: Witten model of case conferences for people with dementia, a narrative approach.
